# Deep learning for protein structure prediction and design—progress and applications

**DOI:** 10.1038/s44320-024-00016-x

**Published:** 2024-01-30

**Authors:** Jürgen Jänes, Pedro Beltrao

**Affiliations:** 1 Institute of Molecular Systems Biology, ETH Zürich, 8093 Zürich, Switzerland; 2 Swiss Institute of Bioinformatics, Lausanne, Switzerland

**Keywords:** AlphaFold2, Structural Bioinformatics, Protein Design, Protein Conformations, Structural Systems Biology, Computational Biology, Structural Biology

## Abstract

Proteins are the key molecular machines that orchestrate all biological processes of the cell. Most proteins fold into three-dimensional shapes that are critical for their function. Studying the 3D shape of proteins can inform us of the mechanisms that underlie biological processes in living cells and can have practical applications in the study of disease mutations or the discovery of novel drug treatments. Here, we review the progress made in sequence-based prediction of protein structures with a focus on applications that go beyond the prediction of single monomer structures. This includes the application of deep learning methods for the prediction of structures of protein complexes, different conformations, the evolution of protein structures and the application of these methods to protein design. These developments create new opportunities for research that will have impact across many areas of biomedical research.

## Abstract (web summary)

Predicting protein structure from sequence information has been a long-standing challenge. This Review discusses recent developments and applications of deep learning-based methods for protein structure prediction and design.

## Graphical abstract

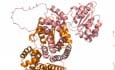

## Introduction

Predicting protein structure from sequence information has been a long-standing challenge in the field of molecular biology. The ability to accurately predict protein structure from sequence information alone would have far-reaching implications for our understanding of biological processes as well as disease and for developing new drugs and therapies.

Historically, one approach to predicting protein structure from sequence information has been homology modeling (Browne et al, 1969). This method relies on the assumption that proteins with similar sequences will have similar structures. By identifying a known protein structure that shares sequence similarity with the target protein, a model of the target protein’s structure can be built. In addition to homology modeling, researchers have also explored the use of co-evolutionary information to predict protein structure. This approach is based on the observation that residues in a protein that are in close spatial proximity often co-evolve (Göbel et al, 1994; Benner and Gerloff, 1991). By analyzing patterns of co-evolution in multiple sequence alignments, it is possible to infer residue-residue contacts and use this information to predict protein structure. The development of prediction methods has progressed steadily over the years including improvements in obtaining residue distance constraints from multiple sequence alignments (Thomas et al, 2008; Dunn et al, 2008; Bartlett and Taylor, 2008; Wang et al, 2017) and in using this information for the prediction of 3D structures (Senior et al, 2020; Xu, 2019; AlQuraishi, 2019). These advances and their historical perspective have been reviewed elsewhere (AlQuraishi, 2021; Laine et al, 2021; Elofsson, 2023) and can be summarized by an increase in usage of neural network models along key parts of the protein structure prediction problem. These developments have led to the notable advance demonstrated by AlphaFold2 that has achieved very high accuracy in sequence-based structure prediction.

In this Review, we will discuss the recent developments and applications of deep learning-based methods for protein structure prediction and design.

## Artificial Intelligence for sequence-based structure prediction

DeepMind showcased the results of AlphaFold2 in the 14th CASP conference in December of 2020. This led to a flurry of activity from different research groups resulting in several end-to-end deep learning models for sequence-based protein structure predictions. These are split into two main groups: alignment-based predictors—e.g., AlphaFold2 (Jumper et al, 2021), RoseTTAFold (Baek et al, 2021), and OpenFold (Ahdritz et al, 2022)—and protein language model-based predictors—including RGN2 (Chowdhury et al, 2022), ESMfold (Lin et al, 2023), OmegaFold (Wu et al, 2022), and EMBER2 (Weissenow et al, 2022). AlphaFold2 takes as inputs a multiple sequence alignment (MSA) and an initial set of pairwise distance measurements that could be optionally initialized via a structural template from a homologous sequence. The architecture is composed of two stages. The first stage processes the MSA and pairwise distances through repeated layers of a transformer-based neural network block dubbed Evoformer. The second stage is a so-called structure module that represents the rotation and translation for each protein residue. Each residue is represented as a triangle of the 3 backbone atoms (nitrogen, alpha-carbon, carbon) and the neural network has learned to move these triangles to the correct place in 3D space to form the predicted structures. The improvements derived from this and other architectures have been reviewed elsewhere (AlQuraishi, 2021) but one critical point is that these models are able to learn how best to extract co-evolution information from a multiple sequence alignment in order to predict distances between residues and the final structure. Removing the possibility of using structural templates has a minimal impact on model performance (Jumper et al, 2021).

RoseTTAFold (Baek et al, 2021) was an explicit attempt to design a model inspired by DeepMind’s presentation of AlphaFold2 at CASP14, at a point when it was unclear if the details of the model were going to be publicly released. The model had a three-track neural network that processes MSA, pairwise distance, and 3D coordinate information simultaneously to produce structure predictions with accuracies that were approaching those of AlphaFold2. A more recent implementation of RoseTTaFold brings its accuracy closer to AlphaFold2 and extends its capabilities to the prediction of RNA and DNA structures (Baek et al, 2024). Finally, OpenFold (Ahdritz et al, 2022) is a reimplementation of AlphaFold2 that has the same architecture but having the code available as well as the data required for re-training. The OpenFold implementation also contains some technical improvements that improve speed and memory usage efficiency. The possibility to retrain this model has already been important to gain insight into what the model has learned and to adapt it for specific applications (as discussed in further detail below). Even though most of these approaches work by integrating co-evolutionary information and structure, there are still differences in their performance. Additional research will determine which specific design decisions are critical for performance.

The major input signal for the models described above is the multiple sequence alignment with the depth of the alignment impacting on the accuracy of the models. However, there are several proteins for which an MSA will not be available. Among others, these include proteins that: have been recently evolved; are rapidly evolving, are designed, or those with rearrangements. Protein language model-based predictors have been developed that aim to replace the need for an MSA with high-dimensional representations of protein sequences that are learned from the protein sequence universe. Protein language models (e.g., epiBERTope (Park et al, 2022), ESM (Rives et al, 2021), ProtTrans (Elnaggar et al, 2022), or ProteinBERT (Brandes et al, 2022)) are neural network models that are trained on predicting masked amino-acids from a very large number of protein sequences. As observed with large language models that have been popularized by chat bots, the simple task of learning masked words has led to models that read and write the language. Similarly, these protein language models capture a representation of protein sequence space that can be passed on to neural network models capable of using this representation to predict protein structure. RGN2, ESMfold, OmegaFold, and EMBER2 are examples of such models that vary in the protein language model used and how these protein sequence representations feed into the structure prediction. All of these models have in common that, in comparison to AlphaFold2, they have a simplified architecture, can run much faster but do not reach the same level of performance when an MSA is available (Elofsson, 2023). These different models have yet to be compared directly on extensive benchmarks but given their speed, they offer the possibility of being applied on a larger scale. As an example, the ESMfold has been used to make predictions for over 700 million proteins (Lin et al, 2023).

Choosing which model to use will depend on the user’s application. For predicting individual structures, the user is better off using AlphaFold2 and putting a larger effort on improving the multiple sequence alignment. For most cases, ColabFold (Mirdita et al, 2022) has modified AlphaFold2 and other methods to run at reduced computational cost with minimal loss in accuracy. Further, ColabFold can fold sequences of up to 1000 residues on Google Colaboratory, without any computational requirements for the user. For individual examples where other approaches are unsuccessful, aggressive sampling as implemented in AFSample (Wallner, 2023) would have the highest chance of success at the expense of significantly more computational resources. Finally, in the absence of an MSA or for very large scale applications, one of the protein language models is likely better suited to the task.

## Opening the black box: what have these models learned

Deep learning methods are complex models with a very large number of parameters which are often described as “black box” models given the difficulty in studying how the models make their predictions and what they have learned. During the training process, the OpenFold team has studied what information their model captured at intermediate steps of the training process (Ahdritz et al, 2022). Independent training runs tended to follow a similar progression where, within the initial steps, the model learned a 1D representation of the structure, followed by 2D and 3D phases of learning that reached reasonably accurate backbone representations. Only then are the representations of the secondary structural elements fully learned, despite the fact that local secondary structure can be predicted even from sequence alone. One of the questions initially raised by the release of AlphaFold2 was the degree of generalization to unseen parts of the protein structural universe. OpenFold addressed this question by training the model on very distinct types of structures, leaving out different protein families or even training on structures composed of single secondary structure elements. Encouragingly, training on these subsets revealed that this architecture is quite robust and can generalize to structures of unseen protein families.

Classical protein structure prediction methods have relied on an energy function to rank possible solutions by considering different energy terms such as the contribution of steric clashes, the formation of hydrogen bonds or electrostatic interactions, etc. To explore if AlphaFold2 may have learned an energy function, Roney and Ovchinnikov used it to rank different related template structures without providing an MSA (Roney and Ovchinnikov, 2022). This analysis indicates that, in the absence of co-evolution signals, AlphaFold2 can rank which structural templates are a better fit to a sequence, suggesting that this model has also learned something akin to an energy function. Additional explorations of these models will be needed to better understand what aspects of biophysics may have been incorporated into them and at what stage of the training process these are acquired.

## Protein structure comparisons empowering evolutionary studies

The development of high-confidence sequence-based structure predictions opens the door for the prediction of structures for large parts of the protein universe. While the unique proteins with solved structures represented in PDB are on the order 100,000, the protein sequences available for analysis are on the order of billions. Currently, the AlphaFold database contains 217 million structures predicted by AlphaFold2 and the ESMfold Metagenomic Atlas contains predicted structures for 772 million proteins. This dramatic increase in the available predicted structures should empower many studies, including the study of diversity of the protein structural universe, the evolution of protein sequences, structures and function, and the potential discovery of novel enzymes. However, analyzing such large numbers of structures also requires the development of highly efficient computational approaches. Even accounting for some high level of redundancy in the sequence databases, such methods would need to be applicable to the scale of tens to hundreds of millions of structures. Examples in this context include the development of efficient methods for pocket comparison (Simonovsky and Meyers, 2020), comparison of protein structures (van Kempen et al, 2023; Durairaj et al, 2020), clustering of protein structures (Barrio-Hernandez et al, 2023) and compression of structural data files (Kim et al, 2023).

The 365,000 structures that were first released in the AlphaFold2 database led to initial attempts of clustering and characterization. A proof-of-principle analysis showed that clustering of these structures based on the similarity of their structural elements could be used to recover groups of known protein families, supporting the use of such approaches for evolutionary studies (Akdel et al, 2022). A protein family analysis of these same structures suggested that around 92% of the predicted domains within this set matched already known superfamilies (Bordin et al, 2023). Recently, an efficient clustering method was used to cluster the 217 million structures in AlphaFold DB, leading to the identification of 2.27 M non-singleton clusters (Barrio-Hernandez et al, 2023). While 31% of these clusters were deemed to represent likely novel structures, these clusters lacking annotations only cover 4% of all proteins in the database. These observations would suggest that the majority of protein structures have, at least partial matches to known protein families. However, the diversity of shapes and functions within each cluster can still be of interest. In addition, these clusters were used for evolutionary studies, identifying cases of remote structural similarity between eukaryotic and prokaryotic structures where sequence-based methods would not easily identify a link.

## Protein complexes and integrative structural modeling

While AlphaFold2 was trained to predict the structures of individual proteins, co-evolutionary information has been used to predict protein-protein interactions since the development of direct coupling analysis algorithms (Weigt et al, 2009). Earlier deep learning methods like Raptor-X (Jing et al, 2020) were making use of protein-interaction contact site predictions for predicting complex structures and it was therefore not unexpected that roseTTAFold and AlphaFold2 could also be applied to this challenge (Mirdita et al, 2022; Akdel et al, 2022; Evans et al, 2021; Ko and Lee, 2021; Bryant et al, 2022a). Multiple independent reports have benchmarked the capacity of AlphaFold2 to predict the structures of complexes and attempted to improve its performance at this task either by improving the correct species pairing of sequences for both proteins in the MSA (Bryant et al, 2022a) or by explicitly training the models on structures of protein complexes (Evans et al, 2021). When comparing against experimental structures, these methods have reported correct predictions of the interface running between 50 to 70% of the cases. In addition, the size of the predicted interface and the confidence estimate for the residues at the interface can be used to rank the predicted models according to an estimated accuracy (Bryant et al, 2022a). It is important to note that this estimated accuracy only relates to cases when it is known that two proteins interact via a direct interface. The prediction accuracy for protein pairs that may or may not include direct interfaces will be lower than the accuracy described above. This can be somewhat circumvented by using the estimated confidence values from the predicted models but should be considered even when deciding what protein pairs to attempt to model. These methods have been applied on a larger scale to predict the structures for previously known protein interactions for *S. cerevisiae*, *B. subtilis*, and human (Humphreys et al, 2021; O’Reilly et al, 2023; Burke et al, 2023), showcasing how these can be applied for example to predict the impact of mutations at protein interfaces. Given the combinatorial nature of protein interactions, using these methods on all known or predicted protein interactions in a species remains resource intensive. The faster protein language-based models, once adapted to this problem, may serve as a useful screening method that could be applied on a very large scale.

**Figure 1:**
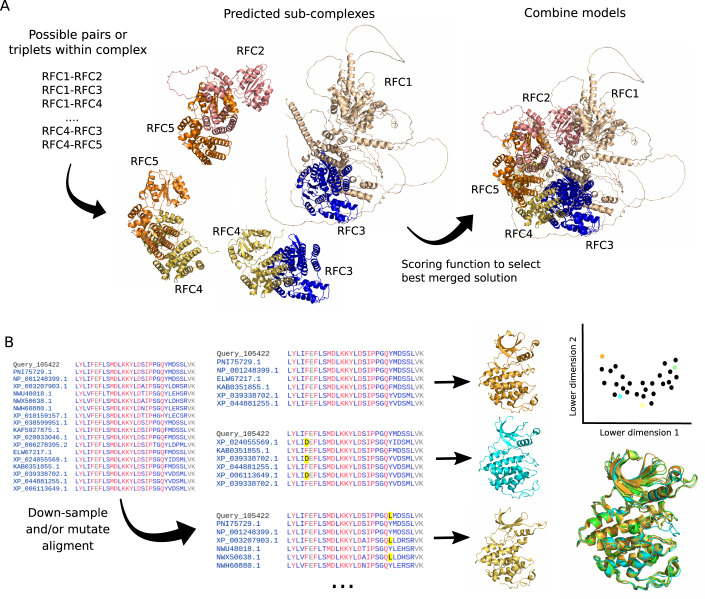
Example applications of AlphaFold2 beyond single protein structure prediction. (**A**) Alphafold2 has shown to be capable of predicting structures for binary protein complexes but predicting structures for larger assemblies remains challenging. A suggested procedure has been to predict the structures for possible sub-complexes and then combine them using superimposition of common subunits (see main text). (**B**) While AlphaFold2 is trained to predict a single conformation, it has been shown that subsampling of the alignment that serves as the main input, can result in the prediction of different conformations that sometimes resemble known conformations.

The prediction of complexes containing larger numbers of proteins remains challenging due to computer memory limitations in these methods. Given these limitations, an approach (see Fig. 1A) has been to predict models for pairs or triplets of proteins in the same complex and develop a strategy to assemble them by superimposition (Burke et al, 2023; Bryant et al, 2022b). These studies have pointed out some limitations, including the need of prior knowledge on the stoichiometry of subunits within the complex and the higher error rates for correct placement of paralogous subunits within the same complex.

The availability of predicted structures for monomers and complexes can also be used as part of pipelines for integrative structural modeling. In integrative modeling of protein complexes, different data modalities are used as restraints in order to score the possible conformations of represented molecules of interest (see review (Ziemianowicz and Kosinski, 2022)). In this application, even the predicted monomer structures are of high interest since they can be used to fit predicted atomic structures for specific proteins in lower-resolution experimental data of larger assemblies. A notable recent example of this was the combination of AlphaFold2-based predictions with cryo-electron tomography data to solve a 70-megadalton model of the human Nuclear Pore Complex (NPC) (Mosalaganti et al, 2022).

## From single structures to ensembles

Proteins are dynamic and can exist in different conformations. Despite this, AlphaFold2 and related methods have been trained to produce a single structural representation for a given protein sequence. Early studies suggested that AlphaFold2 could not predict the structural changes of mutated sequences (Buel and Walters, 2022) or different conformation of proteins that are known to change in structure when bound to a small molecule (Saldaño et al, 2022). However, this was based on multiple runs with the same parameters. Several independent groups then reported success in predicting different conformations by providing, in different runs, an MSA with a smaller set of random sequences from the full alignment (Del Alamo et al, 2022b) (see Fig. 1B). Presumably, such random down-sampling of the alignment may expose co-evolution signals that predispose the prediction towards different conformations. In addition to uniform down-sampling of the alignment, other strategies include the mutation of residues in the alignment that correspond to positions of contact within the structure (Stein and Mchaourab, 2022) or down-sampling of the alignment after clustering the sequences (Wayment-Steele et al, 2024). Down-sampling the MSA by selecting them from a sequence clustering method was shown to substantially improve the prediction of known alternative conformations when compared to uniform random down-sampling. In all cases, the methodology follows a similar strategy: generating a large number of predictions with different perturbations of the alignment; grouping predictions by structural similarity to identify high-confidence predictions that are different from each other; comparing with existing structures or external sources of data.

The prediction of different conformations has now been successfully applied in a number of different systems (see review (Sala et al, 2023)), including transporters and GPCRs (Del Alamo et al, 2022b), different “metamorphic” proteins (KaiB, RfaH, Mad2) (Wayment-Steele et al, 2024), small molecule binding proteins (adenylate cyclase, ribose binding protein, tryptophan synthase) (Casadevall et al, 2022; Stein and Mchaourab, 2022), and proteins with pockets that open in specific conformations (i.e cryptic pockets) (Meller et al, 2023). In one case, the analysis and perturbations of the alignments were used to predict specific mutations that could result in a change of the preferred conformation (Wayment-Steele et al, 2024). Not all attempts at predicting known alternative conformations were successful and it is unclear how the success rate depends on the representation of the different conformation in the training data. Nevertheless, these results indicate that AlphaFold2 and related methods might have the capacity to predict different conformations and can be combined with orthogonal sources data (del Alamo et al, 2022a). However, it is likely that better methodology may be developed, using deep learning models that are specifically trained for the purpose of predicting alternative conformations.

In related efforts, the distributions of inter-residue confidence estimates predicted by AlphaFold2 have been used to construct structural ensembles of intrinsically disordered proteins (Faidon Brotzakis et al, 2023). Alternatively, AlphaFold2 confidence scores have been combined with elastic networks to generate structural ensembles (Jussupow and Kaila, 2023).

## Advances in deep learning methods for protein design

Protein design aims to generate proteins with a pre-determined shape and/or function with great potential for the rational design of enzymes, scaffolds, high-affinity binders, and other functions of biotechnological or therapeutic value. Rational protein design can be seen as the inverse problem of sequence-based protein structure prediction where the objective is to predict a sequence that will have a pre-determined structure or function. Traditionally, this has been achieved by computational protocols that can search through favorable sequences that are ranked according to a physics inspired energy function (review in (Kuhlman and Bradley, 2019)). As for protein structure prediction, deep learning neural network models have been recently applied to dramatically improve on the capacity to design proteins with diverse characteristics (Anand et al, 2022; Strokach et al, 2020; Huang et al, 2022; Anishchenko et al, 2021; Madani et al, 2023; Verkuil et al, 2022; Watson et al, 2023). Recent approaches have adapted similar architectures used for protein structure predictions for the generative task of protein design leading to order-of-magnitude increases in success rates. High experimentally confirmed rates have been reported on the design of proteins with pre-defined backbones (67% success rate measured as solubility and monomeric state) (Verkuil et al, 2022), novel sequences for an existing enzyme family (73%) (Madani et al, 2023), pre-defined oligomerization states with novel proteins (11.5%), novel ion binding proteins (40%) and binders to specific target proteins (18%) (Watson et al, 2023). Importantly, the controllability of the designs has also improved substantially (Watson et al, 2023; Hie et al, 2022) whereby the target protein can be steered by easy to implement user defined constraints. These can, in principle, be tuned to any function for which the target sequence and/or structure can be measured against. It is important to note that these success rates are not strictly comparable due to the differences in defining success for different design tasks and that it remains to be seen if these success rates can be easily replicated in different labs.

## Perspective

AlphaFold2 and related methods have made great progress at predicting structures for well folded single-domains, and made significant advances in other areas such as protein-protein interactions. One major aspect of AlphaFold2’s success lies in combining PDB structures with co-evolutionary signals derived from large protein sequence databases in an end-to-end differentiable way. We think these approaches have the potential to be extended in several directions.

Several mass-spectrometry (MS) assays, such as cross-linking (XL-MS), hydrogen deuterium exchange (HDX-MS), limited proteolysis-coupled mass spectrometry (LiP-MS), can capture information on regions that are in close contact, freely accessible to the solvent or that change in accessibility under some conditions. AlphaFold2 has already been adapted to use in-cell cross-linking information for improved protein modeling (Stahl et al, 2023) and this could be further generalized to consider other sources of constraints that could include the above mentioned MS methods and also constraints from structural methods such as NMR, x-ray crystallography and cryo-EM.

In addition to proteins, it is likely that methods related to AlphaFold may be extendable to other types of molecules. RoseTTAFold has been adapted to predict protein-nucleic acid complexes (Baek et al, 2024). RoseTTAFoldNA has already improved the state of the art despite the low number of available nucleic acid structures. Here, further improvements could be obtained by integrating data from high-throughput protein-nucleic acid profiling experiments. AlphaFold2-related methods cannot yet predict protein-small molecule interactions and docking small ligands into the structure is challenging (Holcomb et al, 2023). A recent method, DiffDock, shows better performance on computationally folded structures (Corso et al, 2022), although overall success rates remain low. Databases such as ChEMBL contain binding information on a non-overlapping set of ligands and targets (Liu et al, 2015), although without structural information. Nevertheless, similarly to the co-evolutionary signal from Uniprot, an end-to-end differentiable pipeline combining PDBbind—a collection of measured binding affinity data for complexes deposited in PDB—with small-molecule binding data could improve protein-small molecule complex predictions.

In addition, the current approaches are limited to predicting a single structure per input sequence (Lane, 2023). Prediction of multiple discrete confirmations is possible in some cases, but complex dynamics such as predicting the exact folding pathways is beyond the reach of current methods (Outeiral et al, 2022). Here, trajectories from molecular dynamics simulations could be used as complementary training input. Early results suggest that this is possible, and can generalize to systems beyond the training set (Janson et al, 2023).

**Figure 2:**
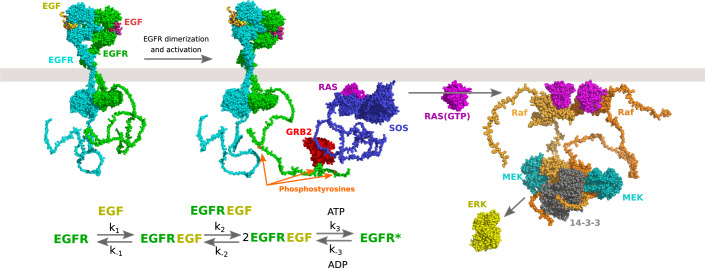
Proteome-wide structural systems biology. Structural details for the initial steps of EFG pathway activation. For representation, the AlphaFold2 predicted structures of pathway components were combined with experimental structures from years of study of this pathway, including PDB ids: 1egf, 1nql, 1m17, 2jwa, 3njp, 2gs6, 1gri, 1xd2, 3ksy, 5p21, 6xi7, 6q0j, 2y4i, 1pme. The AlphaFold2 models help complete the missing protein sequence information not represented in the experimental results, in particular for the long unstructured regions. The example is inspired by similar visualization in PDB-101 (https://pdb101.rcsb.org/learn/exploring-the-structural-biology-of-cancer). It may become possible to use the protein sequences and structures to derive reaction parameters that would allow us to better understand the mechanisms underlying a system of interest.

The combination of improvements in experimental and computational approaches is leading to a revolution in structural biology whereby structural information is expected to cover the full proteomes of key species of interest. In Fig. 2, we combine AlphaFold2 models with experimental structures for proteins in the early steps of EGF pathway activation. In this example, the AlphaFold2 models helped in particular in visualizing the long unstructured regions which give context to those missing sequences. While this model is likely to contain many errors it challenges us to think about the complete atomic details of multi-component cellular processes. While it is clear to us that this expanded structural view of the cell should open many possible research questions it is not yet obvious exactly what the most promising future directions might be. As an example, future developments in this area may include the ability to derive reaction parameters directly from protein sequences/structures in order to model a system of interest. Structural models have always been a means towards better understanding the mechanisms of life. It is up to the research community now to take these advances in bold new directions.
